# Serum *P*-Cresyl Sulfate Level Is an Independent Marker of Peripheral Arterial Stiffness as Assessed Using Brachial-Ankle Pulse Wave Velocity in Patients with Non-Dialysis Chronic Kidney Disease Stage 3 to 5

**DOI:** 10.3390/toxins14040287

**Published:** 2022-04-16

**Authors:** Yu-Chi Chang, Yu-Li Lin, Yu-Hsien Lai, Chih-Hsien Wang, Bang-Gee Hsu

**Affiliations:** 1 Department of Internal Medicine, Hualien Tzu Chi Hospital, Buddhist Tzu Chi Medical Foundation, Hualien 97010, Taiwan; ck11516@gmail.com (Y.-C.C.); nomo8931126@gmail.com (Y.-L.L.); hsienhsien@gmail.com (Y.-H.L.); wangch33@gmail.com (C.-H.W.); 2 Division of Nephrology, Department of Internal Medicine, Hualien Tzu Chi Hospital, Buddhist Tzu Chi Medical Foundation, Hualien 97010, Taiwan; 3 School of Medicine, Tzu Chi University, Hualien 97004, Taiwan

**Keywords:** brachial-ankle pulse wave velocity, *p*-cresyl sulfate, chronic kidney disease, peripheral artery stiffness

## Abstract

*p*-Cresyl sulfate (PCS) is a uremic toxin that causes cardiovascular injury and progression in patients with chronic kidney disease (CKD). Peripheral arterial stiffness (PAS) as measured using the brachial-ankle pulse wave velocity (baPWV) is considered a valuable predictor of cardiovascular event risk in the general population. The study investigated the correlation between serum PCS levels and PAS (baPWV > 18.0 m/s) in 160 patients with stage 3–5 CKD. Liquid chromatography–mass spectrometry was used to assay serum PCS levels. PAS was detected in 54 patients (33.8%), and it was linked to older age, a higher prevalence of hypertension, higher systolic and diastolic blood pressure, higher serum calcium–phosphorus product and PCS levels, and lower height and body weight. Multivariable logistic regression analysis for independent factors associated with PAS illustrated that, in addition to age and diastolic blood pressure, serum PCS levels exhibited an odds ratio (OR) of 1.098 (95% confidence interval = 1.029–1.171, *p* = 0.005). These findings demonstrated that serum PCS levels were associated with PAS among patients with stage 3–5 CKD.

## 1. Introduction

Cardiovascular disease (CVD) is more frequent in patients with chronic kidney disease (CKD) than in the general population [1]. There are several risk factors for CVD, and two main mechanisms are responsible for development of CVD in patients with CKD. Traditional risk factors for CVD, such as essential hypertension, diabetes mellitus, dyslipidemia, and smoking, play an important role in CKD progression because of damage in both large and small vessels [1,2]. Non-traditional risk factors for CVD include vascular calcifications, endothelial inflammation, and marked proteinuria, which result in cardiac dysfunction via valve calcification, impaired activation of the renin–angiotensin–aldosterone system, and myocardial fibrosis with collagen deposition [2].

Arterial stiffness had been recognized as a strong predictor of CVD and mortality independent of traditional risk factors [3,4]. Pulse wave velocity (PWV) is the gold standard for assessing arterial stiffness. PWV in the peripheral arteries, as measured using brachial-ankle PWV (baPWV), exhibited a good correlation with that measured using carotid-femoral PWV (cfPWV). Therefore, baPWV provides a reliable modality for assessing peripheral arterial stiffness (PAS) [4].

*p*-Cresyl sulfate (PCS), generated by protein fermentation mainly in the large intestine, is a protein-bound uremic toxin, which contributes to many biological and biochemical (toxic) effects in patients with CKD [5,6]. In the circulation, PCS mostly binds to albumin in approximately 95% of healthy controls and patients with CKD, and it is eliminated in tubular epithelial cells via secretion [6]. The overgrowth and imbalance of gastrointestinal microbiota and worsening glomerular filtration rates cause PCS accumulation in plasma among patients with CKD [6,7]. High serum PCS levels result in vascular calcification, arterial stiffness, endothelial dysfunction attributable to inflammation, and an elevated risk of CVD, which is the leading cause of mortality in patients with CKD [8,9].

It is known that increased serum PCS levels are associated with an elevated CVD risk among patients with CKD. In response to the lack of significant data of the association between serum PCS levels and PAS, we investigated the correlation between serum PCS content and PAS as measured via baPWV in patients with clinically significant stage 3–5 CKD.

## 2. Results

Table 1 presents the baseline characteristics of the total 160 patients with non-dialysis stage 3–5 CKD categorized into control (*n* = 106) and PAS (*n* = 54 (33.8%)) groups. Compared with the control group, the proportion of non-dialysis stage 3–5 CKD patients with hypertension was significantly higher in the PAS group (*p* = 0.026). In addition, compared with the control group, the PAS group was typified by older age (*p* < 0.001), shorter height (*p* = 0.033), lower body weight (*p* = 0.012), and higher systolic blood pressure (SBP, *p* < 0.001), diastolic blood pressure (DBP, *p* = 0.008), serum calcium–phosphorus product levels (*p* = 0.030), and PCS levels (*p* = 0.038). By contrast, there were no significant differences in sex, the prevalence of comorbid conditions, including chronic glomerulonephritis and diabetes mellitus, or the CKD stage between the groups.

Serum concentrations of PCS across CKD stages 3–5 were depicted in Supplementary Figure S1, which showed significantly increasing PCS levels with progressing CKD stage (*p* for trend < 0.001).

Adjustment for the factors significantly associated with PAS (hypertension, age, body mass index (BMI), SBP, DBP, eGFR, calcium–phosphorus product, and PCS) in multivariable logistic regression analysis revealed that serum PCS levels (odds ratio (OR) = 1.098, 95% confidence interval (CI) = 1.029–1.171, *p* = 0.005), age (OR = 1.105, 95% CI = 1.055–1.159, *p* < 0.001), and DBP (OR = 1.058, 95% CI = 1.002–1.118, *p* = 0.043) were the independent predictors of PAS in patients with stage 3–5 CKD (Table 2).

**Figure 1:**
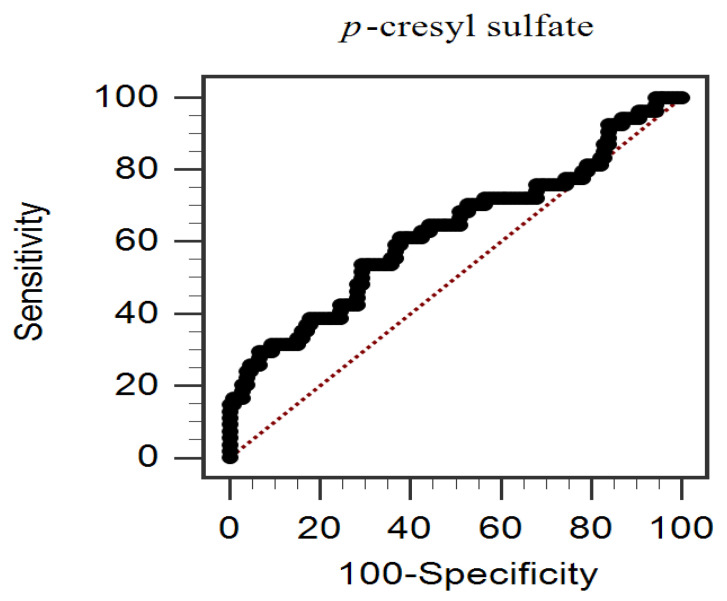
The receiver operating characteristic curve analysis of serum total *p*-cresyl sulfate levels to predict peripheral arterial stiffness in chronic kidney disease patients.

Receiver operating characteristic (ROC) curve analysis indicated that the area under the curve (AUC) for predicting PAS was 0.628 (95% CI = 0.531–0.725, *p* = 0.0096, Figure 1). The best cut-off of PCS level was ≥20.49 mg/L, which provided 53.7% sensitivity and 70.8% specificity. In addition, a near-vertical line of the ROC curve for the initial portion indicated that, when high cut-offs (≥34.92 mg/L) were used, 100% specificity could be achieved without making any false positive error.

The correlation and linear regression of brachial-ankle pulse wave velocity and clinical variables are shown in Table 3 and Table 4. Left and right baPWV were positively correlated with age, hypertension, height, body weight, SBP, DBP, serum calcium–phosphorus product levels, and logarithmically transformed PCS (log-PCS). Moreover, serum phosphorus levels were positively correlated with right baPWV (*p* = 0.040). In multivariate stepwise linear regression analysis, age (*β* = 0.455, adjusted coefficient of determination (r^2^) = 0.165, *p* < 0.001), body weight (*β* = −0.134, adjusted r^2^ = 0.013, *p* = 0.038), DBP (*β* = 0.380, adjusted r^2^ = 0.155, *p* < 0.001), and higher log-PCS (*β* = 0.204, adjusted r^2^ = 0.045, *p* = 0.002) were significantly correlated with left baPWV, whereas age (*β* = 0.478, adjusted r^2^ = 0.193, *p* < 0.001), body weight (*β* = −0.149, adjusted r^2^ = 0.018, *p* = 0.021), DBP (*β* = 0.342, adjusted r^2^ = 0.126, *p* < 0.001), and higher log-PCS (*β* = 0.198, adjusted r^2^ change = 0.042, *p* = 0.002) were significantly correlated with right baPWV.

## 3. Discussion

In our CKD participants, who were divided into PAS and control groups, 33.8% of them were classified as having PAS. The major findings of the present study were that old age, elevated DBP, and high serum PCS levels are independent predictors of PAS among non-dialysis patients with stage 3–5 CKD. Furthermore, age, DBP, and log-PCS were positively associated with baPWV, whereas body weight was negatively associated with baPWV.

Age and blood pressure (SBP and DBP) are considered the two most important factors in the pathophysiology of arterial stiffness [10,11]. Overall, complicated and multiple mechanisms are involved in the development of arterial stiffness, such as endothelial dysfunction, dysregulated remodeling of elastin and collagen, arterial calcification, and decreases in the lumen diameter with a premature return of the reflected wave in late systole [2,4,12]. Large arteries are centrally and peripherally located, and they exhibit different characteristics. Specifically, central arteries have more elastic components, whereas peripheral arteries are muscular and thus stiffer in nature. Recent studies reported that the age-related increases in PAS are much less significant than in the central arteries in patients with hypertension [4,13,14] and in the general population [15,16]. Several prior studies reported the relationships of central arterial stiffness with age and blood pressure [2,3,4,11,12,13], and our study identified similar effects on PAS in patients with CKD.

Overweight and obesity are strongly associated with advanced arterial stiffness and pronounced risks of CVD and mortality [17,18]. The assessment of overweight and obesity requires different parameters in addition to simple body weight, such as BMI, the waist–hip ratio (WHR), waist circumference (WC), and the visceral fat area (VFA). A recent meta-analysis reported significantly higher arterial stiffness measured by cfPWV, baPWV, and the augmentation index in overweight subjects [19]. However, another study found that baPWV was associated with VFA and WHR but not with BMI and WC, indicating that arterial stiffness might be highly correlated with abdominal obesity rather than overall obesity [20]. Interestingly, a cross-sectional study of 578 Chinese participants, including 288 overweight (25 ≤ BMI < 28 kg/m^2^) or obese (BMI ≥ 28 kg/m^2^) subjects (49.8%), illustrated that PAS, as measured via baPWV, increased with BMI in the overweight/obese group, whereas BMI was negatively associated with baPWV in the whole cohort [21]. Therefore, these results highlight the presence of the obesity paradox [21]. In our study, body weight was negatively associated with baPWV, and the participants in the control group exhibited greater body weight than those in the PAS group.

Elevated serum calcium–phosphorus product levels are associated with vascular calcification, and they contribute to arterial stiffness with increased pulse pressure [22,23]. In patients with CKD, dysregulated mineral metabolism causes long-term hyperphosphatemia and transient hypercalcemia and further activates vascular smooth muscle cells (VSMCs) to induce vascular calcification via osteogenic/chondrogenic differentiation, apoptosis, and extracellular matrix degradation [24,25]. Both fibrosis of VSMCs and activation of fibroblasts induced by high serum calcium and phosphorus levels result in increased peripheral vascular resistance [26]. In this study, high serum calcium–phosphorus product levels were found in the PAS group, in line with the conclusions of recent studies, and the positive associations of serum calcium–phosphorus product levels with elevated SBP and DBP accorded with the results of Anser et al. [22].

Although our previous study noted a positive association between serum PCS levels and central arterial stiffness in patients on hemodialysis [9], the exact association between serum PCS levels and PAS remains unclear because of a lack of research. Lin et al. revealed that PCS levels were correlated with vascular access failure and thrombosis rates among patients receiving hemodialysis, and PCS plays an important role in peripheral artery disease given that serum PCS accumulation induces endothelial dysfunction and smooth muscle cell hyperplasia [27]. An observational study of patients on hemodialysis reported that increased serum PCS levels are positively correlated with carotid atherosclerosis severity and progression through reactive-oxygen-species-mediated apoptosis in endothelial cells [28]. The mechanisms of PCS inducing arterial calcification include the osteogenic/chondrogenic transdifferentiation of VSMCs via the upregulation of bone-related genes (e.g., alkaline phosphatase, RUNX2, osteopontin) and downregulation of smooth muscle genes (e.g., α-smooth muscle actin, smooth muscle 22α) [29,30]. In the present study, the serum PCS level was an independent marker of PAS that was positively associated with baPWV in non-dialysis patients with stage 3–5 CKD.

The main limitations of the study were its cross-sectional and single-center design and small sample size. The result might not be applicable to the general conditions in the CKD population, which exhibits considerable heterogeneity. In addition, patients with non-CKD and early-stage CKD (stage 1–2) were not included, and gut microbiota was not assessed in this study. Meanwhile, the exact mechanism by which PCS could influence the development of PAS remains unclear, and some biomarkers potentially mediating the link between PCS and arterial stiffness, such as matrix Gla protein and osteopontin, were not available. Therefore, additional longitudinal studies with large samples and diverse designs are needed to clarify these findings.

## 4. Conclusions

In addition to older age and elevated DBP as the traditional risk factors, increased serum PCS levels were independently predictive of PAS in patients with non-dialysis stage 3–5 CKD. The baPWV values also positively associated with serum PCS levels in this study. These findings suggested that PCS may influence the development of PAS among patients with stage 3–5 CKD, but the mechanism remains to be elucidated.

## 5. Materials and Methods

### 5.1. Patients

Non-dialysis CKD patients older than 18 years with regular follow-up at the nephrology outpatient department of a medical center in Hualien, Taiwan, were invited to participate in the study between January 2016 and December 2016. CKD was defined by two measurements of the estimated glomerular filtration rate (eGFR) calculated using the Chronic Kidney Disease Epidemiology Collaboration equation, in which the measurements were separated by an interval of >3 months [31]. eGFRs of less than 60 mL/min/1.73 m^2^ were considered indicative of CKD, and the patients were classified according to the Kidney Disease Outcomes Quality Initiative criteria as follows: stage 3 CKD, eGFR = 30–59 mL/min/1.73 m^2^; stage 4 CKD, eGFR = 15–29 mL/min/1.73 m^2^; and stage 5 CKD, eGFR < 15 mL/min/1.73 m^2^ [32]. All patients received multidisciplinary CKD care that focused on dietary salt and protein restriction and the avoidance of nephrotoxic agents. The present study was approved by the Research Ethics Committee, Hualien Tzu Chi Hospital, Buddhist Tzu Chi Medical Foundation (IRB108-96-B). Participants with medical illnesses, including malignancies, chronic inflammatory diseases, heart failure, and chronic obstructive pulmonary disease, at the time of blood sampling, and those who refused to provide informed consent for the study, were excluded. Among 210 patients screened, we finally enrolled 160 participants. Each patient provided written informed consent before participation.

The information regarding smoking status, comorbid diseases (diabetes mellitus, hypertension, and glomerulonephritis), and drugs used (angiotensin-receptor blocker, β-blocker, calcium-channel blocker, α-adrenergic blocker, statin, and fibrate) was collected from the electronic medical record.

### 5.2. Anthropometric Analysis

After an overnight fast, the anthropometric variables were measured in the morning. Height and body weight were recorded to the nearest 0.5 cm and 0.5 kg, respectively. BMI was calculated as weight (kg) divided by height-squared (m^2^) [9].

### 5.3. Biochemical Investigations

Serum levels of fasting glucose, blood urea nitrogen, creatinine, albumin, total cholesterol, triglycerides, low-density lipoprotein cholesterol, blood urea nitrogen, creatinine, total calcium, and phosphorus were determined by an autoanalyzer (Siemens Advia 1800; Siemens Healthcare GmbH, Henkestr, Erlangen, Germany) immediately after the blood samples were obtained from participants after a fast for 10 h [9,33].

### 5.4. Determination of Serum Total PCS Levels by High-Performance Liquid Chromatography–Mass Spectrometry

We used a Waters e2695 high-performance liquid chromatography system containing a mass spectrometer (ACQUITY QDa, Waters Corporation, Milford, MA, USA) to determine the serum total PCS levels according to our previous study [9]. Mass spectrometry was performed in full scan ranges of 50–450 m/z for positive-ion modes and 100–350 m/z for negative-ion modes, respectively, to monitor the participants’ compound (PCS: 187.0 m/z). The procurement and analysis of all examinations were performed using the Empower^®^ 3.0 software (New York, NY, USA).

### 5.5. BaPWV Measurements

Patients were placed in the supine position and rested for 10 min after blood sampling. Then, four pneumatic cuffs connected to both plethysmographic and oscillometric sensors were wrapped around both upper arms and ankles to assess baPWV using a volume plethysmography apparatus (VaSera VS-1000, Fukuda Denshi Co. Ltd., Tokyo, Japan) as described previously [33]. baPWV > 18 m/s was considered a statistically adequate cut-off for individuals with a high risk of CVD and subjects with hypertension as per the suggestion in the Physiological Diagnosis Criteria for Vascular Failure Committee of Japan [34]. As a result, we defined high PAS group as a left or right baPWV exceeding 18.0 m/s in the present study.

### 5.6. Statistical Analysis

Assuming that PAS is present in one-third of participants, with a mean PCS level of 16 ± 9 mg/L and an odds ratio of 1.07 per 1 mg/L increase in PCS, a total of at least 136 patients should be enrolled to achieve a power of 90% (α-level 0.05) [9].

The normality of continuous variables was evaluated by Kolmogorov–Smirnov test. Continuous data were expressed as the mean ± standard deviation for those with normal distribution and as the median with interquartile range for those with non-normal distribution. Between-group comparisons were performed using Student’s independent *t*-test or the Mann–Whitney *U*-test, as appropriate. Categorical variables were expressed as numbers with percentages and compared using the χ^2^ test. Multivariable logistic regression analysis (“Enter” model) was applied to the significant variables between control group (baPWV ≤ 18.0 m/s) or PAS group (baPWV > 18.0 m/s) (adopted factors were hypertension, age, BMI, SBP, DBP, eGFR, calcium–phosphorus product, and PCS). The ROC curve was used to calculate the AUC and determine serum PCS levels predictive of PAS in patients with stage 3–5 CKD. The correlation analysis was performed using Pearson’s correlation (for continuous variables) or Spearman’s rank correlation coefficient (for categorical variables). Non-normally distributed continuous variables were logarithmically transformed when applied to the Pearson correlation. Multivariate stepwise linear regression analyses were used to analyze the variables predicting left or right baPWV in patients with stage 3–5 CKD. SPSS software for Windows (version 19.0; SPSS, Chicago, IL, USA) was used for all statistical analyses. *p* < 0.05 indicated statistical significance.

## Figures and Tables

**Table 1:** Baseline characteristics of the 160 chronic kidney disease patients with control group (baPWV ≤ 18.0 m/s) or peripheral arterial stiffness group (baPWV > 18.0 m/s).

Characteristics	All Patients (*n* = 160)	Control Group (*n* = 106)	PAS Group (*n* = 54)	*p* Value
Age (years)	66.90 ± 11.81	63.91 ± 11.19	72.78 ± 10.84	<0.001 *
Height (cm)	158.37 ± 9.09	159.46 ± 9.40	156.22 ± 8.12	0.033 *
Body weight (kg)	65.33 ± 14.43	67.37 ± 14.99	61.34 ± 12.45	0.012 *
Body mass index (kg/m^2^)	25.92 ± 4.64	26.35 ± 4.75	25.06 ± 4.35	0.095
Left baPWV (m/s)	16.68 ± 3.08	14.86 ± 1.72	20.24 ± 1.79	<0.001 *
Right baPWV (m/s)	16.59 ± 2.93	15.07 ± 2.24	19.58 ± 1.42	<0.001 *
SBP (mmHg)	143.23 ± 17.71	139.23 ± 18.19	151.07 ± 13.79	<0.001 *
DBP (mmHg)	82.43 ± 10.85	80.83 ± 10.42	85.57 ± 11.06	0.008 *
Total cholesterol (mg/dL)	158.64 ± 39.99	159.94 ± 42.10	156.07 ± 35.72	0.564
Triglyceride (mg/dL)	119.50 (90.00–163.50)	119.00 (91.00–166.25)	123.50 (83.00–152.25)	0.854
LDL-C (mg/dL)	89.55 ± 33.42	92.13 ± 35.03	84.48 ± 29.66	0.172
Fasting glucose (mg/dL)	109.50 (98.00–135.75)	106.00 (97.75–130.75)	115.00 (98.75–139.00)	0.498
Blood urea nitrogen (mg/dL)	31.00 (24.00–45.50)	29.50 (23.75–44.00)	32.50 (23.50–48.50)	0.691
Creatinine (mg/dL)	1.70 (1.40–2.58)	1.70 (1.30–2.50)	1.90 (1.40–2.93)	0.320
eGFR (mL/min)	33.75 ± 15.93	35.48 ± 15.52	30.38 ± 16.32	0.055
Albumin (g/dL)	4.1 (3.9–4.4)	4.1 (3.9–4.4)	4.1 (3.7–4.3)	0.059
Total calcium (mg/dL)	8.93 ± 0.47	8.88 ± 0.39	9.03 ± 0.58	0.058
Phosphorus (mg/dL)	3.81 ± 0.78	3.73 ± 0.70	3.96 ± 0.92	0.084
Ca × P product (mg^2^/dL^2^)	34.00 ± 7.14	33.13 ± 6.14	35.71 ± 8.60	0.030 *
Total *p*-cresyl sulfate (mg/L)	18.86 (15.14–24.76)	17.87 (14.91–23.26)	21.60 (15.97–29.45)	0.008 *
Female, *n* (%)	82 (51.3)	53 (50.0)	29 (53.7)	0.658
Diabetes mellitus, *n* (%)	68 (42.5)	45 (42.5)	23 (42.6)	0.987
Hypertension, *n* (%)	115 (71.9)	70 (66.0)	45 (83.3)	0.021 *
Glomerulonephritis, *n* (%)	38 (23.8)	24 (22.6)	14 (25.9)	0.644
Current smoking, *n* (%)	24 (15.0)	16 (15.1)	8 (14.8)	0.963
ARB use, *n* (%)	86 (53.8)	58 (54.7)	28 (51.9)	0.731
β-blocker use, *n* (%)	40 (25.0)	26 (24.5)	14 (25.9)	0.847
CCB use, *n* (%)	77 (48.1)	48 (45.3)	29 (53.7)	0.313
α-adrenergic blocker use, *n* (%)	19 (11.9)	11 (10.4)	8 (14.8)	0.412
Statin use, *n* (%)	80 (50.0)	57 (53.8)	23 (42.6)	0.181
Fibrate use, *n* (%)	24 (15.0)	16 (15.1)	8 (14.8)	0.963
CKD stage 3, *n* (%)	89 (55.6)	64 (60.4)	25 (46.3)	0.221
CKD stage 4, *n* (%)	41 (25.8)	25 (23.6)	16 (29.6)	
CKD stage 5, *n* (%)	30 (18.8)	17 (16.0)	12 (24.1)	

Values for continuous variables are given as mean ± standard deviation and tested by Student’s *t*-test; variables not normally distributed are given as median and interquartile range and tested by Mann–Whitney U-test; values are presented as number (%), and analysis was performed using the chi-square test. PAS, peripheral arterial stiffness; baPWV, brachial-ankle pulse wave velocity; SBP, systolic blood pressure; DBP, diastolic blood pressure; LDL-C, low-density lipoprotein cholesterol; eGFR, estimated glomerular filtration rate; Ca × P product, calcium–phosphorus product; ARB, angiotensin-receptor blocker; CCB, calcium-channel blocker; CKD, chronic kidney disease. * *p* < 0.05 was considered statistically significant.

**Table 2:** Multivariable logistic regression analysis of the factors correlated with peripheral arterial stiffness among 160 chronic kidney disease patients.

Variables	Odds Ratio	95% CI	*p* Value
Total *p*-cresyl sulfate (mg/L)	1.098	1.029–1.171	0.005 *
Age (years)	1.105	1.055–1.159	<0.001 *
Diastolic blood pressure (mmHg)	1.058	1.002–1.118	0.043 *
Hypertension (present)	0.723	0.212–2.466	0.604
Body mass index (kg/m^2^)	0.923	0.838–1.107	0.106
Systolic blood pressure (mmHg)	1.023	0.984–1.064	0.250
Ca × P product (mg^2^/dL^2^)	1.067	0.987–1.153	0.105
eGFR (mL/min)	1.032	0.996–1.069	0.080

Data analysis was performed using the multivariate logistic regression analysis (adopted factors: hypertension, age, body mass index, systolic blood pressure, diastolic blood pressure, estimated glomerular filtration rate, calcium–phosphorus product, and *p*-cresyl sulfate). CI, confidence interval; eGFR, estimated glomerular filtration rate; Ca × P product, calcium–phosphorus product. * *p* < 0.05 was considered statistically significant. Post hoc power of total *p*-cresyl sulfate = 99.8%.

**Table 3:** Correlation and linear regression of left brachial-ankle pulse wave velocity and clinical variables among 160 patients with chronic kidney disease stage 3–5.

Variables	Left Brachial-Ankle Pulse Wave (m/s)				
	Simple Correlation		Multivariable Linear Regression		
	*r*	*p* Value	Beta	Adjusted R^2^ Change	*p* Value
Female	0.053	0.502	—	—	—
Diabetes mellitus	−0.045	0.571	—	—	—
Hypertension	0.258	0.001 *	—	—	—
Age (years)	0.413	<0.001 *	0.455	0.165	<0.001 *
Height (cm)	−0.192	0.015 *	—	—	—
Body weight (kg)	−0.201	0.011 *	−0.134	0.013	0.038 *
Body mass index (kg/m^2^)	−0.118	0.137	—	—	—
Systolic blood pressure (mmHg)	0.403	<0.001 *	—	—	—
Diastolic blood pressure (mmHg)	0.317	<0.001 *	0.380	0.155	<0.001 *
Total cholesterol (mg/dL)	0.010	0.901	—	—	—
Log-triglyceride (mg/dL)	−0.014	0.863	—	—	—
LDL-C (mg/dL)	−0.038	0.634	—	—	—
Log-glucose (mg/dL)	0.013	0.870	—	—	—
Log-BUN (mg/dL)	0.002	0.983	—	—	—
Log-creatinine (mg/dL)	0.056	0.482	—	—	—
eGFR (mL/min)	−0.121	0.126	—	—	—
Log-albumin (g/dL)	−0.077	0.333			
Total calcium (mg/dL)	0.121	0.128	—	—	—
Phosphorus (mg/dL)	0.131	0.098	—	—	—
Ca × P product (mg^2^/dL^2^)	0.163	0.039 *	—	—	—
Log-PCS (mg/L)	0.289	<0.001 *	0.204	0.045	0.002 *

Data of triglyceride, glucose, BUN, creatinine, albumin, and PCS levels showed skewed distribution and therefore were log-transformed before analysis. Analysis of data was performed using the Pearson or Spearman correlation and multivariable stepwise linear regression analysis (adopted factors were hypertension, age, height, body weight, systolic blood pressure, diastolic blood pressure, Ca × P product, and log-PCS). HDL-C, high-density lipoprotein cholesterol; LDL-C, low-density lipoprotein cholesterol; BUN, blood urea nitrogen; eGFR, estimated glomerular filtration rate; Ca × P product, calcium–phosphorus product; PCS, *p*-cresyl sulfate. * *p* < 0.05 was considered statistically significant.

**Table 4:** Correlation and linear regression of right brachial-ankle pulse wave velocity and clinical variables among 160 patients with chronic kidney disease stage 3–5.

Variables	Right Brachial-Ankle Pulse Wave (m/s)				
	Simple Correlation		Multivariable Linear Regression		
	*r*	*p* Value	Beta	Adjusted R^2^ Change	*p* Value
Female	0.061	0.445	—	—	—
Diabetes mellitus	−0.058	0.464	—	—	—
Hypertension	0.264	0.001 *	—	—	—
Age (years)	0.446	<0.001 *	0.478	0.193	<0.001 *
Height (cm)	−0.205	0.009 *	—	—	—
Body weight (kg)	−0.222	0.005 *	−0.149	0.018	0.021 *
Body mass index (kg/m^2^)	−0.139	0.080	—	—	—
Systolic blood pressure (mmHg)	0.384	<0.001 *	—	—	—
Diastolic blood pressure (mmHg)	0.272	<0.001 *	0.342	0.126	<0.001 *
Total cholesterol (mg/dL)	0.047	0.556	—	—	—
Log-triglyceride (mg/dL)	−0.018	0.821	—	—	—
LDL-C (mg/dL)	−0.012	0.880	—	—	—
Log-glucose (mg/dL)	0.003	0.970	—	—	—
Log-BUN (mg/dL)	0.015	0.852	—	—	—
Log-creatinine (mg/dL)	0.050	0.526	—	—	—
eGFR (mL/min)	−0.134	0.092	—	—	—
Log-albumin (g/dL)	−0.123	0.121			
Total calcium (mg/dL)	0.117	0.139	—	—	—
Phosphorus (mg/dL)	0.163	0.040 *	—	—	—
Ca × P product (mg^2^/dL^2^)	0.192	0.015 *	—	—	—
Log-PCS (mg/L)	0.411	<0.001 *	0.198	0.042	0.002 *

Data of triglyceride, glucose, BUN, creatinine, albumin, and PCS levels showed skewed distribution and therefore were log-transformed before analysis. Analysis of data was performed using the Pearson or Spearman correlation and multivariable stepwise linear regression analysis (adopted factors were hypertension, age, height, body weight, systolic blood pressure, diastolic blood pressure, phosphorus, Ca × P product, and log-PCS). HDL-C, high-density lipoprotein cholesterol; LDL-C, low-density lipoprotein cholesterol; BUN, blood urea nitrogen; eGFR, estimated glomerular filtration rate; Ca × P product, calcium–phosphorus product; PCS, *p*-cresyl sulfate. * *p* < 0.05 was considered statistically significant.

## Data Availability

The data presented in this study are available on request from the corresponding author.

## Supplementary Materials

The following are available online at https://www.mdpi.com/article/10.3390/toxins14040287/s1. Figure S1: Serum concentrations of PCS across CKD stages 3–5.
