# Supplementary material for: Serum P-Cresyl Sulfate Level Is an Independent Marker of Peripheral Arterial Stiffness as Assessed Using Brachial-Ankle Pulse Wave Velocity in Patients with Non-Dialysis Chronic Kidney Disease Stage 3 to 5
Source: Toxins (Basel). 2022 Apr 16;14(4):287. doi: 10.3390/toxins14040287 (PMC9032097; doi:10.3390/toxins14040287)
Supplement: Supplementary file 1 [file toxins-14-00287-s001.zip › toxins-1666719-supplementary.pdf]

Supplementary Materials: Serum *P*-Cresyl Sulfate Level Is an Independent Marker of Peripheral Arterial Stiffness as Assessed Using Brachial-Ankle Pulse Wave Velocity in Patients with Non-Dialysis Chronic Kidney Disease Stage 3 to 5

Yu-Chi Chang, Yu-Li Lin, Yu-Hsien Lai, Chih-Hsien Wang and Bang-Gee Hsu

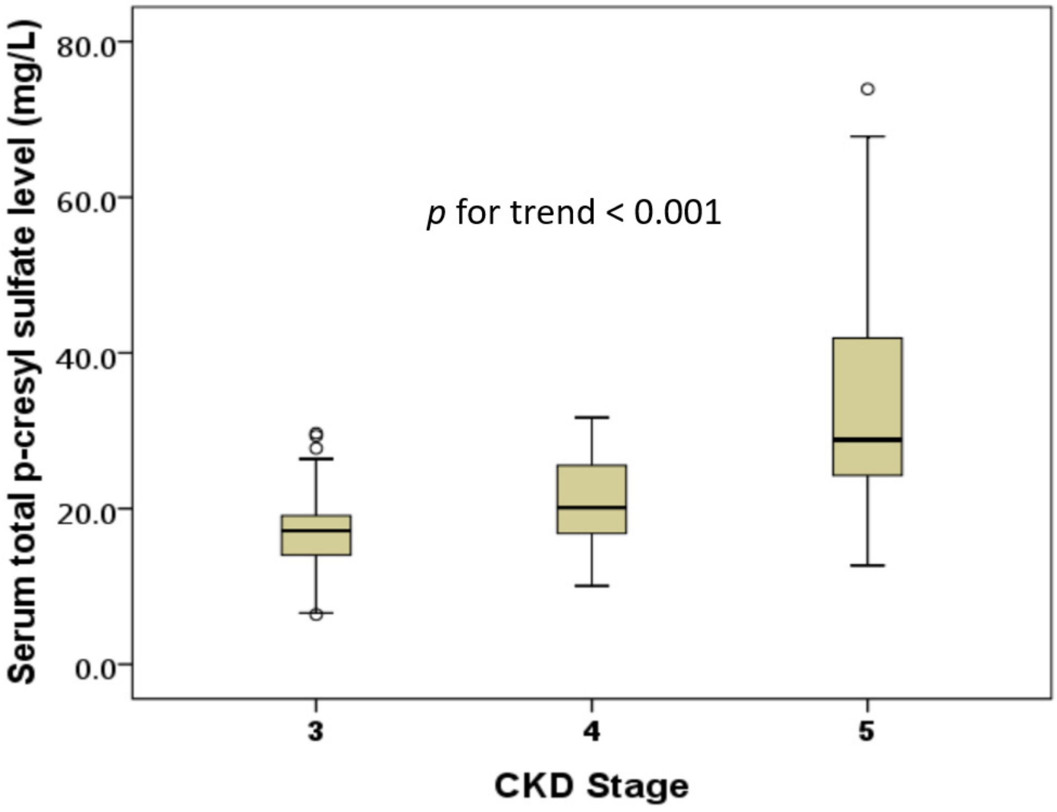

Figure S1. Serum total *p*-Cresyl sulfate levels in different stages of chronic kidney disease in this study.

Toxins **2022**, *14*, 287. https://doi.org/10.3390/toxins14040287 www.mdpi.com/journal/toxins
